# Sulfoquinovosyl diacylglycerol is required for dimerisation of the *Rhodobacter sphaeroides* reaction centre-light harvesting 1 core complex

**DOI:** 10.1042/BCJ20240125

**Published:** 2024-06-26

**Authors:** Elizabeth C. Martin, Adam G.M. Bowie, Taylor Wellfare Reid, C. Neil Hunter, Andrew Hitchcock, David J.K. Swainsbury

**Affiliations:** 1 Plants, Photosynthesis and Soil, School of Bioscience, University of Sheffield, Sheffield, U.K.; 2 School of Biological Sciences, University of East Anglia, Norwich, U.K.

**Keywords:** light harvesting, lipids, photosynthesis, RC-LH1, reaction centre, *Rhodobacter sphaeroides*

## Abstract

The reaction centre-light harvesting 1 (RC-LH1) core complex is indispensable for anoxygenic photosynthesis. In the purple bacterium *Rhodobacter* (*Rba.*) *sphaeroides* RC-LH1 is produced both as a monomer, in which 14 LH1 subunits form a C-shaped antenna around 1 RC, and as a dimer, where 28 LH1 subunits form an S-shaped antenna surrounding 2 RCs. Alongside the five RC and LH1 subunits, an additional polypeptide known as PufX provides an interface for dimerisation and also prevents LH1 ring closure, introducing a channel for quinone exchange that is essential for photoheterotrophic growth. Structures of *Rba. sphaeroides* RC-LH1 complexes revealed several new components; protein-Y, which helps to form the quinone channel; protein-Z, of unknown function and seemingly unique to dimers; and a tightly bound sulfoquinovosyl diacylglycerol (SQDG) lipid that interacts with two PufX arginine residues. This lipid lies at the dimer interface alongside weak density for a second molecule, previously proposed to be an ornithine lipid. In this work we have generated strains of *Rba. sphaeroides* lacking protein-Y, protein-Z, SQDG or ornithine lipids to assess the roles of these previously unknown components in the assembly and activity of RC-LH1. We show that whilst the removal of either protein-Y, protein-Z or ornithine lipids has only subtle effects, SQDG is essential for the formation of RC-LH1 dimers but its absence has no functional effect on the monomeric complex.

## Introduction

The purple phototrophic bacterium *Rhodobacter* (*Rba.*) *sphaeroides* contains hundreds of specialised chromatophore vesicles, ∼50 nm in diameter, the number of which responds to the incident light intensity [1–3]. The major membrane complexes of the chromatophore comprise the reaction centre-light harvesting 1 (RC-LH1) core complex, the peripheral light harvesting 2 (LH2) antenna complex, the cytochrome (cyt) *bc*_1_ complex, and ATP synthase [4]. Of these, cryogenic electron microscopy (cryo-EM) structures of cyt *bc*_1_ [5], LH2 [6] and both the monomeric and dimeric forms of RC-LH1 [7–10] have recently been determined. Within the chromatophore, LH2 complexes each bind a circular array of bacteriochlorophyll (BChl) and carotenoid pigments that absorb light and transfer the energy via the LH1 complex to the RC, where the energy is transiently stored as a charge separation [11–13]. Light-driven reduction of quinone to quinol at the RC is followed by passage of quinol across the LH1 ring [13] and diffusion to a cyt *bc*_1_ complex, where the quinol is oxidised through operation of the Q-cycle [14–16]. The cyt *bc*_1_ complexes reside in locally lipid-rich domains [4,17] located within a few nanometres of the RC-LH1 complexes [18]. The catalytic mechanism of the cyt *bc*_1_ complex reduces cytochrome *c*_2_ [11,19], which returns to the photo-oxidised RC, completing the cyclic electron transfer pathway [11,20]; the Q-cycle mechanism also releases protons into the chromatophore lumen, generating a proton-motive force that is utilised by ATP synthase to drive the production of ATP to power cellular metabolism [20,21].

In *Rba. sphaeroides*, the RC consists of 3 subunits (L, H and M) and is surrounded in a fixed stoichiometry by a C-shaped LH1 ring containing 14 pairs of α and β transmembrane polypeptides, each of which binds 2 BChls and 2 carotenoids [7–10,22,23]. Closure of the LH1 ring is prevented by a single copy of the PufX subunit, which precludes the insertion of further LH1 subunits to maintain the open complex [7–10,24–28]. One copy of the recently discovered protein-Y (also referred to as PufY or protein-U) subunit sits between the RC and the LH1 ring, creating the RC_3_-LH1_14_-XY complex (using the naming convention suggested by Swainsbury et al. [13]) and maintaining a separation that allows quinones and quinols to diffuse freely to and from the RC Q_B_ site [7–10].

The many structural variations of purple bacterial RC-LH1 complexes are reviewed in [13]. Most RC-LH1 complexes are monomeric. Some have a closed LH1 ring, as in *Rhodospirillum rubrum* [29,30] and *Thermochromatium* *tepidum* [31], while others have an incomplete ring held open by extra scaffolding subunits such as PufX (*Rba. veldkampii*) [32], protein-W (*Rhodopseudomonas palustris*) [33–35], or a transmembrane helix associated with the cytochrome subunit (*Roseiflexus castenholzii*) [36,37]. In this context, RC-LH1 complexes in *Rba. sphaeroides* are unusual because they form dimers in which 28 LH1 subunits form an S-shaped assembly around 2 RCs, creating a path for energy transfer between the 2 halves of the complex. This arrangement provides an elegant energy-conservation mechanism that allows an LH1 excited state access to a second RC if the first is already undergoing a charge separation [38,39]. In addition, the dimeric complex is bent, with the two monomers held at an angle of 152° [8–10,23], which imposes curvature on the membranes and is partly responsible for the spherical shape of the chromatophore vesicles [1,8–10,23,40–43]. This property is unique to a few close relatives of *Rba. sphaeroides*, which were recently reclassified into the *Cereibacter* genus [44]. To ensure consistency with the previous literature we will refer to these species as belonging to the *Cereibacter* subgroup but continue to use the historical species names for *Rba. sphaeroides* and its relatives throughout this manuscript. Mutants of *Rba. sphaeroides* that lack PufX are not only unable to grow photoheterotrophically but also no longer form dimers [7,24,25,28,45,46].

**Figure 1.**
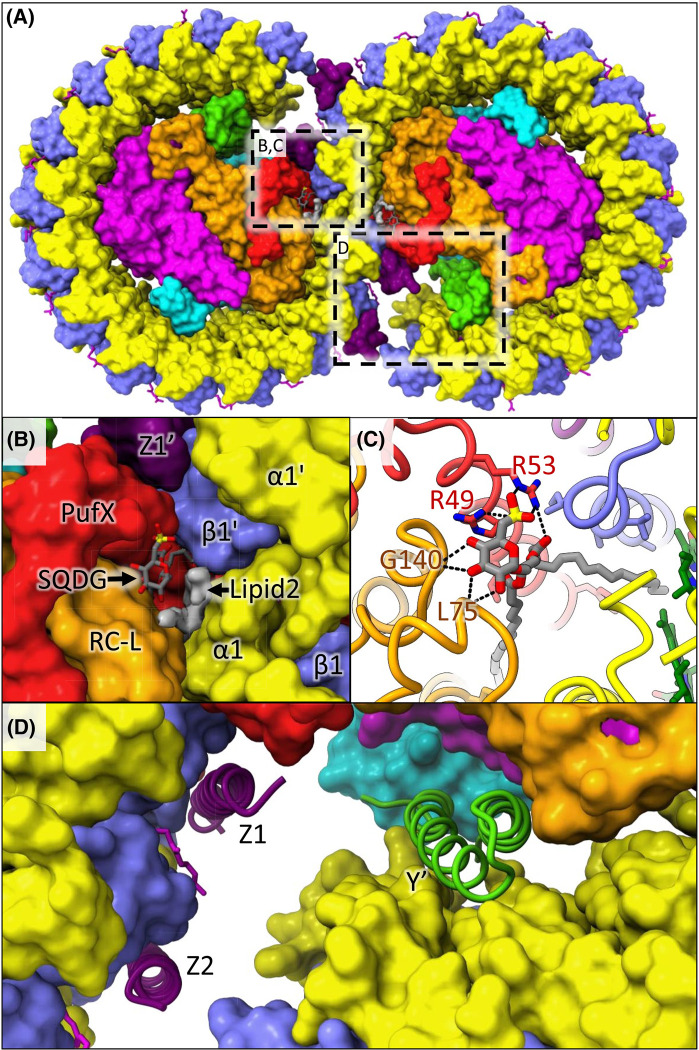
Structure of the dimeric *Rba. sphaeroides* RC-LH1 core complex. (**A**) Surface view of the complex from the lumenal (periplasmic) face. The RC subunits are shown in orange (RC-L), magenta (RC-M) and cyan (RC-H). LH1 subunits are in yellow (α) and blue (β). Additional subunits are in red (PufX), green (protein-Y) and purple (protein-Z). Lipids and cofactors are shown in stick representation in green (BChl), magenta (carotenoids) and grey (SQDG). Unassigned density for lipid 2 is shown as a grey surface. Dashed boxes illustrate areas enlarged in panels (**B**), (**C**) and (**D**). (**B**) Enlarged view highlighting one of the SQDGs and lipid 2 bound at the dimer interface. The subunits from the left monomer (PufX, RC-L, α1 and β1), and those from the right monomer (α1′, β1′ and Z1′) are labelled. (**C**) A further enlarged view of the SQDG lipid with the protein in ribbon representation. Hydrogen bonds between the SQDG head group and PufX Arg49 and Arg53, and the backbone of RC-L residues Leu75 and Gly140 are shown. (**D**) Enlarged view of the two protein-Z subunits bound to the left monomer (Z1 and Z2, purple) and protein-Y of the right monomer (Y′, green) in ribbon representation. The rest of the protein is in surface representation.

Recent cryo-EM structures of the monomeric and dimeric forms of *Rba. sphaeroides* RC-LH1 have revealed further components in the complex beyond PufX (Figure 1). Protein-Y forms a hydrophobic hairpin structure that lies between the inside surface of LH1α 13 and 14 and the RC. Genetic removal of protein-Y results in the formation of an incomplete LH1 ring with as few as 11 α and 10 β subunits [9,10]. This suggests that in addition to promoting the access of quinones to the RC Q_B_ site, protein-Y also provides a binding site for the LH1 subunits at the edge of the LH1 array, ensuring a gap that is correctly positioned to facilitate rapid diffusion of quinone and quinol between the RC and the external quinone pool [13]. Four copies of a second novel transmembrane polypeptide, protein-Z, were also identified in the dimeric structure, but are absent from the monomer, suggesting an as yet undetermined role unique to the dimer [8]. The presence of both protein-Y and protein-Z was confirmed by mass spectrometry of RC-LH1 complexes from wild-type *Rba. sphaeroides* [7,8]. The dimeric structure also revealed the presence of two lipids, the first of which was confidently assigned as sulfoquinovosyl diacylglycerol (SQDG) based on clear density of its distinctly shaped sulfonic acid head group. The other lipid was less well defined but was proposed to be an ornithine lipid [8]. The SQDG lipid was found to bridge the two halves of the dimer, interacting with both the RC-L subunit and the critical Arg49 and Arg53 residues of PufX in one monomer, and an LH1 β subunit in the other (Figure 1A–C). This interaction would explain why mutation of either Arg49 or Arg53 to Lys disrupts dimer formation [46] as this would prevent the interaction between SQDG and PufX that appears to hold the two monomers together.

In this study, we investigate the roles of SQDG, ornithine lipids, and the Y and Z subunits of the RC-LH1 complex by generating a series of mutants deficient in synthesis of specific lipids or lacking the genes that encode protein-Y or protein-Z. The effects of these mutations on RC-LH1 dimerisation, RC activity and photoheterotrophic growth have been characterised, providing new insights into these recently identified components of the RC-LH1 core complex.

## Results

### Generation and verification of strains lacking SQDG and ornithine lipids

**Figure 2.**
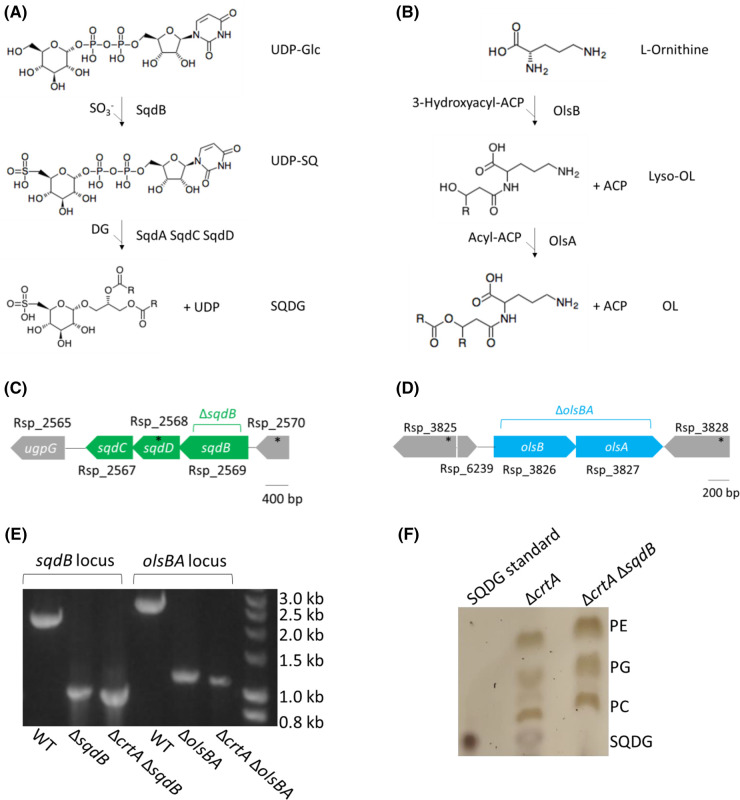
Generation of strains deficient in SQDG or ornithine lipids. (**A**) Sulfoquinovosyl diacylglycerol (SQDG) is synthesised in two steps. The first step is the addition of sulphite to UDP-glucose (UDP-Glc) to produce UDP-sulfoquinovose (UDP-SQ) by SqdB. Next, diacylglycerol (DG) is added and UDP is removed by SqdA, C and D to produce SQDG [47]. (**B**) Ornithine lipids are synthesised by the acyltransferases OlsB and OlsA, which sequentially add a 3-hydroxyacyl group then an acyl group to L-ornithine using 3-hydroxyacyl-ACP and acyl-ACP as substrates, respectively [48,49]. (**C**) Structure of the *Rba. sphaeroides sqdBDC* operon. The region labelled Δ*sqdB* (Rsp_2569) was removed to abolish SQDG biosynthesis. (**D**) Structure of the *Rba. sphaeroides olsBA* operon. The labelled region spanning *olsB* (Rsp_3826) and *olsA* (Rsp_3827) was removed to prevent OL biosynthesis. (**E**) Agarose gel of ethidium bromide-stained PCR products showing size differences for the amplified regions spanning the *sqdB* and *olsBA* genes showing a clear reduction in size in the knockout strains relative to the wild type. (**F**) TLC plate showing loss of SQDG in the Δ*sqdB* strain. By comparison to a SQDG standard, SQDG is present in membranes from Δ*crtA* but not Δ*crtA* Δ*sqdB*, confirming the loss of SQDG biosynthesis in the absence of SqdB. An uncropped image showing the standards for the other lipids is shown in Supplementary Figure S1.

As shown in Figure 2A, SQDG synthesis from UDP-glucose requires four enzymes: SqdB, SqdA, SqdC and SqdD [47]; we deleted the *sqdB* (Rsp_2569) gene from the *sqdBDC* operon to prevent the first step of SQDG biosynthesis (Figure 2C). Two enzymes, OlsA and OlsB, are required to produce ornithine lipids from L-ornithine [48,49] (Figure 2B). The *olsB* (Rsp_3826) and *olsA* (Rsp_3827) genes are arranged as an operon in which the 3′ end of *olsB* and the 5′ end of *olsA* overlap (Figure 2D); this gene pair was deleted to prevent ornithine lipid production. Previous work has shown that the deletion of *crtA* influences the levels of RC-LH1 dimer formation [50]. We therefore deleted *sqdB* and *olsBA* in both a wild-type background and in a strain lacking the spheroidene monooxygenase (Δ*crtA*) to better visualise any effect of lipid content on the monomer-dimer ratio. The successful deletion of either *sqdB* or *olsBA* from the genome of the wild type or Δ*crtA* mutant is shown in Figure 2E.

**Figure 3.**
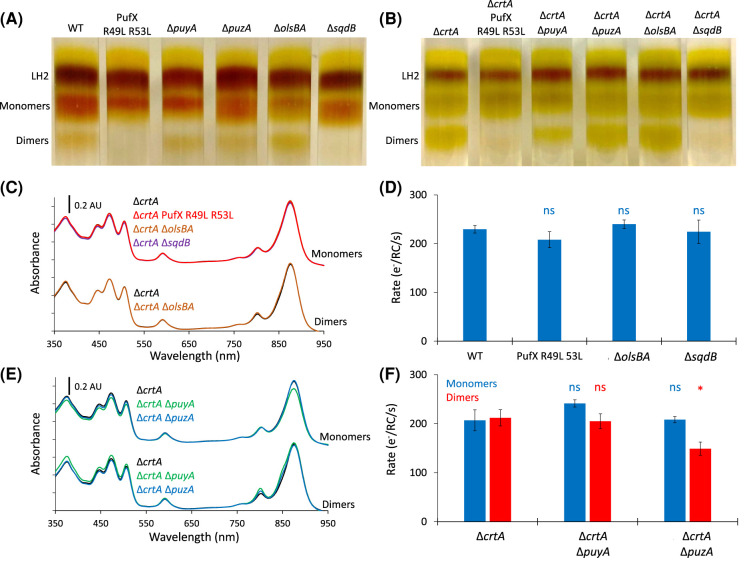
The effect of removing protein-Y, protein-Z, SQDG or ornithine lipids on the RC-LH1 monomer: dimer ratio and activity. (**A,B**) Separation of LH2, RC-LH1 monomer and RC-LH1 dimer complexes from detergent-solubilised membranes on sucrose step gradients. (**A**) The wild-type strain (WT), a control strain that does not produce dimeric RC-LH1 (PufX R49L R53L), a strain lacking protein-Y (*ΔpuyA*), a strain lacking protein-Z (*ΔpuzA*), a strain that cannot produce ornithine lipids (*ΔolsBA*), and a strain that cannot produce SQDG (*ΔsqdB*). (**B**) Sucrose gradients for the strains in (**A**) in a background that is also deficient in the *crtA* gene encoding spheroidene monooxygenase (*ΔcrtA*). Two further repeats of the gradients in panels (**A)** and (**B)** are shown in Supplementary Figure S2. (**C**) UV/Vis/NIR spectra for monomer and dimer bands harvested from gradients for the *ΔcrtA* strain, the RC-LH1 dimer-deficient *ΔcrtA* PufX R49L R53L strain, and the *ΔcrtA ΔolsBA* and *ΔcrtA ΔsqdB* strains. The LH1 maximum is at 873 nm. (**D**) Turnover assays for monomeric complexes from WT, PufX R49L R53L, *ΔolsBA* and *ΔsqdB* mutants. Rates show moles of cyt *c*_2_ oxidised (reported as electrons) per second per mole of RC during illumination using an 810 nm LED of a solution containing 0.05 μM RC-LH1 and 5 μM cyt *c*_2_. Unpaired two-tailed t-tests (for three technical replicates) were performed relative to rates for monomeric or dimeric WT complexes where ns denotes a p-value >0.05. (**E**) UV/Vis/NIR spectra for monomer and dimer bands harvested from gradients for the *ΔcrtA* strain, and those also harbouring the *ΔpuyA* and *ΔpuzA* mutations. The LH1 maximum is at 873 nm, with low levels of LH2 contamination contributing to the slight shoulder at 850 nm. (**F**) Turnover assays for monomeric and dimeric RC-LH1 complexes from the *ΔcrtA*, *ΔcrtA ΔpuyA* and *ΔcrtA ΔpuzA* strains under the same conditions as in (**D**) except the RC concentration, which was 0.01 μM. A p-value <0.05 is denoted by * and ns denotes a p-value >0.05.

Thin-layer chromatography (TLC) confirmed the loss of SQDG when *sqdB* is deleted (Figure 2F) but ornithine lipids were not detectable, preventing their analysis using this method. Under photoheterotrophic conditions, growth of the strains unable to produce SQDG or ornithine lipids was indistinguishable from strains with unaltered lipid biosynthesis (Supplementary Figure S3), and the UV/Vis/NIR spectra of isolated chromatophore membranes from these strains were also very similar (Supplementary Figure S4). Therefore, removal of SQDG or ornithine lipids did not result in obvious phenotypes with respect to either growth or spectral features.

### The loss of SQDG prevents the formation of dimeric RC-LH1 core complexes

The monomer: dimer ratio in membranes from different strains of *Rba. sphaeroides* was determined using rate-zonal centrifugation of sucrose gradients. The removal of SQDG in wild-type and Δ*crtA* strains results in a complete loss of observable dimer formation, whereas the removal of ornithine lipids has no discernible effect upon the monomer to dimer ratio of RC-LH1 complexes (Figure 3A,B). The absence of SQDG-free RC-LH1 dimers supports the assignment of SQDG in our cryo-EM structure and highlights the essential role of this lipid for RC-LH1 dimer formation in *Rba. sphaeroides*. The absence of dimers from a control strain known to only produce monomers, in which the PufX Arg49 and Arg53 residues that hydrogen-bond the SQDG headgroup are replaced with leucines [8,46], further supports the role of SQDG in dimerisation. The normal growth of the cells, and near-identical absorbance spectra of complexes from strains unable to produce SQDG and ornithine lipids (Supplementary Figures S3 and S4 and Figure 3C), suggests that RC-LH1 monomers are properly assembled without these lipids. Introducing *sqdB* to the Δ*sqdB* strain on a replicative plasmid restores some formation of dimers, whilst introducing the complete *sqdBDC* operon fully restores dimer formation to wild-type levels (Supplementary Figure S5A). To test whether SQDG could induce dimerisation of fully assembled monomeric complexes, we attempted to dimerise monomeric RC-LH1 complexes from the Δ*sqdB* strain by the addition of SQDG. The monomer band harvested from a 20–25% discontinuous gradient of Δ*sqdB* membranes was incubated with or without 0.05% w/v SQDG for 24 h, but no significant dimer formation was seen (Supplementary Figure S5B). The inability of exogenously added SQDG to induce dimerisation suggests that the lipid must be fully integrated during the *in vivo* assembly pathway in order to create the wide range of interactions that stabilise the RC-LH1 dimer. These include a series of hydrogen bonds with the backbone of the RC-L subunit, the salt bridge complex with PufX Arg49 and Arg53, and hydrophobic contacts with the transmembrane region of the opposing LH1β1 subunit and the BChl1 macrocycle on the other side of the complex [8].

To test whether SQDG and ornithine lipids affect RC-LH1 activity *in vitro*, we monitored the light-driven oxidation rate of cytochrome *c*_2_ by monomeric and dimeric complexes in the presence of ubiquinone-2 (an analogue of the native substrate, ubiquinone-10 with a shortened isoprene tail). In monomeric RC-LH1 complexes from the wild-type background, there was no significant difference in turnover rates between the Δ*sqdB* and Δ*olsBA* mutants and equivalent complexes with both lipids, demonstrating no impairment of assembly or function in the absence of either lipid (Figure 3D). Therefore, we can conclude that whilst deletion of *sqdB* and therefore removal of SQDG biosynthesis precludes dimer formation, there is no functional effect on the monomeric complex.

### The absence of protein-Y or protein-Z does not prevent dimer formation

As the *puyA* ORF (Rsp_7571) encoding protein-Y is isolated in the genome, we were able to excise it without disturbing upstream or downstream genes, leaving behind a sequence encoding 6 residues in the genome of the unmarked Δ*puyA* mutant strain. The gene encoding protein-Z is located within the Rsp_2385 open reading frame on chromosome 1 (1 014 511–1 014 819) but is transcribed in the opposite direction [8]. Most of this gene was deleted to make a Δ*puzA* strain, with just the sequence encoding seven residues left intact in the genome. Confirmation of successful knockouts are shown in Supplementary Figure S6.

Rate-zonal centrifugation of solubilised chromatophores from these strains show the removal of neither protein-Y nor protein-Z prevents the formation of dimers (Figure 3A,B). Absorption spectra of each monomer band shows a small but distinct decrease in absorbance at 873 nm in strains lacking protein-Y (Figure 3E). This observation agrees with the finding that the absence of protein-Y results in monomers missing some α and β LH1 polypeptides, thus they have fewer BChls per core complex [9,10]. The dimer bands all have near-identical UV/Vis/NIR spectra, suggesting all dimers contain the same number of α and β polypeptides. The monomer to dimer ratio is similar to wild type in strains lacking either protein-Y or protein-Z, which appear to have no significant role in dimer formation.

Activity assays show that the rate of cytochrome *c*_2_ oxidation by wild-type RC-LH1 monomers and dimers are almost identical, and that rates for both forms of the Δ*puyA* RC-LH1 complex are similar, even when accounting for the slight reduction in absorbance at 873 nm (Figure 3E,F). The activity of the monomeric complexes from the Δ*puzA* strain were found to be similar to the monomeric wild-type complexes, but the activity of the dimeric complexes lacking protein-Z was slightly reduced (*P* = 0.03), suggesting it may have a small, but not essential, functional role exclusive to the dimer (Figure 3F).

### SQDG, PufX, protein-Y and protein-Z in other *Rhodobacter* species

**Figure 4.**
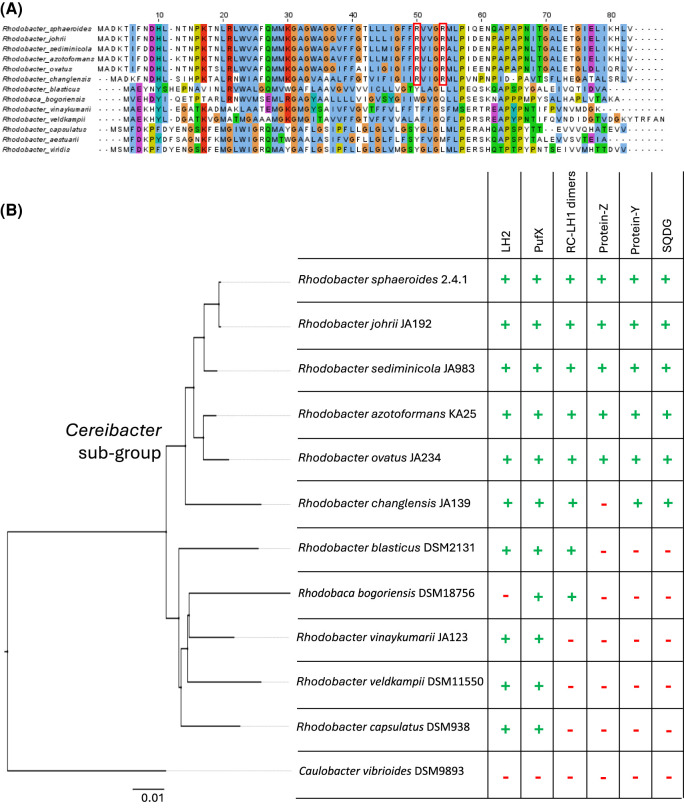
Phylogenetic analysis of the proteins involved in RC-LH1 dimer formation. (**A**) Sequence alignment of PufX polypeptides. Red outlined boxes indicate the two arginine residues that bind SQDG in *Rba. sphaeroides*. (**B**) 16S rRNA phylogeny of selected PufX-producing species of the *Rhodobacteriales* order for which the oligomeric state of their RC-LH1 complex is known. The root of the *Cereibacter* subgroup is labelled. *Caulobacter vibrioides*, a non-photosynthetic alphaproteobacterium, is included as an outgroup to root the tree. The table shows the presence (green + symbols) or absence (red — symbols) of genes encoding the LH2 complex, PufX, protein-Y and protein-Z, and whether the species produces dimeric RC-LH1 complexes and SQDG (the latter inferred by the presence of the *sqdB* gene).

Aligning PufX sequences shows that the two arginine residues that bind SQDG, Arg49 and Arg53, are universally conserved amongst the *Rhodobacter* species within the *Cereibacter* subgroup (Figure 4). All of these species also contain genes for SQDG biosynthesis (*sqdBDC*), suggesting RC-LH1 complexes in these species may form dimers with PufX and SQDG in a similar way to *Rba. sphaeroides*. *Rba. azotoformans* and *Rba. changelensis* form dimeric RC-LH1 complexes [51], but this has yet to be verified for other members of the *Cereibacter* sub-group. The Arg residues are not conserved beyond the *Cereibacter* group and a BLAST search for either *sqdB*, *C* or *D* in the species lacking the PufX Arg residues found no results*.* We would not expect species with monomeric RC-LH1 complexes to require SQDG, but it is interesting to note that there are two species with dimeric RC-LH1 (*Rhodobaca bogoriensis* [52] and *Rba. blasticus* [53]) that contain neither the Arg49 and Arg53 residues in PufX nor the genes for SQDG biosynthesis. Further exploration is required to see if a different lipid, perhaps with a different mode of binding to PufX, is fulfilling the role of SQDG in dimerisation in these organisms.

We identified sequences for protein-Y in all species within the *Cereibacter* subgroup, and they have extremely high sequence homology that exceeds 94%, except for *Rba. changlensis*, where this value is only 38% (see Figure 4 and Supplementary Figure S7 for sequence alignments). Protein-Z could also be found in all species in the *Cereibacter* subgroup except for *Rba. changlensis*. The fact that the *Rba. changlensis* PufX sequence is also distinct from the rest of the *Cereibacter* subgroup, and its clear separation in phylogenetic trees generated from 16S rRNA sequences (Figure 4), indicates it may be a more distant relative of *Rba*. *sphaeroides*. We also note that sequence homology was quite high in the first 30 residues of protein-Z, corresponding with the 31 residues resolved in the cryo-EM structure [8], but very low in the rest of the sequence, suggesting function may be limited to the N-terminus.

Sequences with homology to *Rba. sphaeroides* protein-Y and protein-Z were not found outside of the *Cereibacter* subgroup. It may be that if proteins are fulfilling the same role, they are not similar enough to be found through searches using sequence homology. This may be the case for the RC-LH1 complex from *Rhodobaca bogoriensis*; structural analysis reveals a protein in a position similar to that adopted by protein-Y in *Rba. sphaeroides* [54], but the protein identified in this study bears no homology to protein-Y.

## Discussion

### The roles of PufX, SQDG and OL in RC-LH1 dimer formation

PufX has long been known as a unique component of the RC-LH1 complexes of *Rhodobacter* species [24] (recently renamed to *Cereibacter* [44]). In species that produce dimeric forms of the RC-LH1 complex, PufX is essential for mediating the interaction between pairs of RC-LH1 complexes to form the S-shaped dimers [8–10,23,41,55]. As such, PufX has been the subject of intense investigation to understand how and why certain forms of this polypeptide drive RC-LH1 dimer formation whilst others from closely related species do not [51]. Various parts of the PufX polypeptide have been investigated as potential points of contact between monomers, including a conserved GxxxG motif [27,56], the N-terminal 12 amino acids [26], and a pair of Arg residues at positions 49 and 53 (*Rba. sphaeroides* numbering) that are unique to those PufX polypeptides in dimer forming species [46]. It was not until the determination of high-resolution structures of RC-LH1 dimers that the structure of PufX was modelled with sufficient detail to elucidate the interactions that bring the two RC-LH1 monomers together [8–10]. These structures revealed that the N-terminal amino acids of PufX interact with the cytoplasmic end of the PufX polypeptide from the neighbouring monomer [9,10]. Additionally, a short ‘LWVAF’ motif close to the cytoplasmic surface of the membrane mediates a direct protein-protein interaction between the two PufX proteins, and PufX residues 44–67 bind to the lumenal surface of the RC-L subunit. Conversly, the GxxxG motif plays no role in bringing together opposing PufX polypeptides.

The large distance between Arg49 and Arg53 in one PufX and those in the opposing monomer was a surprising discovery because these residues are known to be essential for dimer formation. In strains where these Arg residues are replaced with other amino acids [46], and in the PufX Arg49Leu Arg53Leu mutant strain we used to determine the structure of the monomeric complex [7], the formation of RC-LH1 dimers is abolished. Careful inspection of the cryo-EM map for the RC-LH1 dimer revealed density consistent with an SQDG lipid for which the head group was hydrogen bonded to Arg49 and Arg53. One of the lipid hydrocarbon tails extends between PufX and the L-subunit within the RC-LH1 monomer, and the second tail extends across the dimer interface towards the opposing monomer [8] (Figure 1). The specific nature of the headgroup binding, which could not reasonably accommodate an alternative lipid, and the requirement for Arg49 and Arg53 for dimer formation led us to speculate that the SQDG lipid itself is essential for dimer formation, which we confirmed here via disruption of the SQDG biosynthesis pathway.

In contrast with our findings with SQDG, the removal of ornithine lipids had no observable effect on dimer formation. This suggests that ‘lipid 2’ (Figure 1 and Qian et al. [8]) may not be an ornithine lipid, or that the lipid bound in this position can be substituted for an alternative. Because the density was ambiguous, possibly because of more disorder for lipid 2 relative to SQDG, we are still unable to assign it reliably. We also cannot determine whether lipid 2 is required for dimer formation. However, its close association with SQDG and its position in a cavity between RC-LH1 monomers would suggest that the presence of a lipid in this location plays an important role.

Surprisingly, removal of SQDG or ornithine lipids had no observable effect on photoheterotrophic growth (Supplementary Figure S3) or on the activity of the RC-LH1 complexes (Figure 3D). This is consistent with previous observations that disruption of dimer formation does not produce a discernible phenotype under laboratory conditions [46]. This finding also suggests that specific protein-SQDG or protein-ornithine lipid interactions are not essential in other membrane protein complexes required for growth under photoheterotrophic conditions, although they may be important under other growth modes, as is the case for ornithine lipid-deficient strains of *Rba. capsulatus* [49]. SQDG is abundant in whole chromatophore membranes and in the annular lipids of complexes extracted using styrene-maleic acid copolymer nanodiscs [17,57,58], so it appears that bound SQDG or ornithine lipid can often be substituted with other lipids, with the clear exception of the SQDG at the RC-LH1 dimer interface. This is not the first example of essential, ordered lipids being present in RC-LH1 complexes. Most purple bacteria RCs are known to bind a conserved cardiolipin, the disruption of which (via mutation of interacting residues) adversely effects RC thermostability [59–61]. There are also many lipids observed between the RC and LH1 ring, many of which are common to all structurally resolved RC-LH1 complexes and interact via conserved residues on the RC and LH1 α subunits [13,33]. Therefore, this study, and those mentioned above, highlight the important role of protein-lipid interactions, which are often unresolved in reported RC-LH1 structures.

### The roles of protein-Y and protein-Z in dimer formation and RC activity

It had long been assumed that the RC-LH1 complexes of *Rba. sphaeroides* contained six discrete proteins (RC-L, RC-M, RC-H, PufX, LH1 α and LH1 β), so the discovery of protein-Y and protein-Z in the cryo-EM structures was unexpected [7–10]. Protein-Y was annotated as a hypothetical protein in UniprotKB, whilst protein-Z was unannotated in *Rba. sphaeroides* 2.4.1 (see [8]). An immediate question posed by the discovery of these new subunits was whether they are required for RC-LH1 dimer formation and photoheterotrophic growth of *Rba. sphaeroides*.

The removal of protein-Y does not inhibit dimer formation, as indicated by the similar relative abundance of the monomeric and dimeric complexes (Figure 3A,B). However, the spectra of the monomeric complex show a decrease in LH1 absorbance at 873 nm relative to RC absorbance at 803 and 760 nm (Figure 3E), suggesting a lowered LH1 antenna size. Cao et al. [9] and Tani et al. [10] recently determined the structure of RC-LH1 lacking protein-Y (referred to as PufY and protein-U in these studies) and found that, whilst both monomer and dimer complexes still form, the final subunits of the LH1 antenna fail to assemble or are dissociated during protein purification. Our findings are consistent with the generation of the same complexes with a smaller LH1 antenna. Spectroscopic analysis by Thwaites et al. [62] showed that removal of protein-Y does not result in a marked change in the rate and efficiency of energy transfer from LH1 to the RC relative to the wild-type complex. Because protein-Y is distant from the dimer interface and does not interact with PufX or SQDG, it is reasonable that its removal does not affect the ability of the complex to form dimers. Photoheterotrophic growth of the strains unable to produce protein-Y was similar to the wild type (Supplementary Figure S3), which suggests that the loss of four LH1 subunits has a negligible effect on light harvesting under our laboratory conditions. We previously suspected that protein-Y serves to maintain a channel for efficient quinone exchange, which seems at-odds with our *in vitro* assays. However, loss of the terminal LH1 subunits creates a larger opening in the LH1 ring, which may compensate for the absence of protein-Y and the reduction in light-harvesting capacity. Such a loss of capacity would likely not influence growth rates under the illumination conditions used for our experiments.

In strains that lack protein-Z there was no observable effect on dimer formation nor on photoheterotrophic growth. The activity of monomeric complexes was similar to the wild type, which was expected because monomeric RC-LH1 does not appear to bind protein-Z [7–10]. However, the dimeric complexes that lack protein-Z had slightly lowered activity when compared with wild-type dimers (Figure 3F). This suggests that protein-Z may act to ensure the quinone exit channel at the dimer interface is kept open by preventing the complex reverting to the closed state observed by Cao et al. [9]. We note that these assays were only performed in triplicate under a single condition and so are not exhaustive, with further studies required to elucidate the full impact of removing protein-Z. Despite the small reduction in activity in purified RC-LH1 dimers lacking protein-Z we did not find an effect on growth rates under our laboratory growth conditions (Supplementary Figure S3).

### The evolution of RC-LH1 dimers is synergistic with the presence of SQDG, protein-Y and protein-Z

To further understand the roles of SQDG, protein-Y and protein-Z, we investigated the genomes of other bacteria capable of forming dimeric RC-LH1 complexes. By searching the relevant databases, we found that the species reclassified into the *Cereibacter* group, all of which are either known to form dimers or we predict will form dimers, appear to contain the genes for SQDG biosynthesis, PufX with SQDG-binding Arg residues, and protein-Y. Additionally, most of these species also contain protein-Z. Conversely, *Rhodobacter* species that produce monomeric complexes do not appear to contain any of these proteins. Whilst the phylogenetic analysis we performed is not exhaustive, we are able to speculate that the formation of RC-LH1 dimers in the *Cereibacter* subgroup evolved with the ability to synthesise SQDG, and the evolution of a variant of PufX that could bind the lipid head group. Subsequently, protein-Y was recruited to maintain efficient quinone diffusion. Finally, protein-Z was recruited to lock the complete RC-LH1 dimer in its final conformational state. Exceptions to this are the dimeric RC-LH1 complexes from more distantly related *Rhodobaca bogoriensis* and *Rba. blasticus,* which appear not to produce SQDG and seem to be more closely related to the monomer-producing *Rba. capsulatus* and *Rba. veldkampii* than the *Cereibacter* subgroup. At the time of writing, the dimeric structure of the *Rhodobaca bogoriensis* complex is published as a preprint [54] and the co-ordinates are not available in the PDB. However, unlike *Rba. sphaeroides* its LH1 is composed of 15 LH1 subunits and it does not produce LH2, so it may have achieved RC-LH1 dimer formation by a unique mechanism that warrants further investigation.

## Materials and methods

### Generation of strains and plasmids

The strains used in this study are detailed in Table 1, primer sequences are provided in Supplementary Table S1, and plasmid information is given in Supplementary Table S2. Genomic modifications were made using the pK18mob*sacB* plasmid as previously described [63]. Briefly, the target genes were deleted by amplifying ∼400 bp regions upstream and downstream of each gene and joining them by overlap extension PCR, yielding a sequence lacking most of the coding region but leaving the start and stop codons intact and in frame to guard against interference with downstream genes upon genomic modification. The resulting PCR fragments were digested with EcoRI and HindIII and ligated into pK18mob*sacB* cut with the same restriction enzymes. The resulting sequence-verified plasmids were transformed into *Escherichia coli* S17-1 and then conjugated into either wild-type or Δ*crtA Rba. sphaeroides.* Correctly modified strains were isolated following sequential selection with kanamycin (30 µg/ml) and counter-selection with sucrose (10% w/v) and the modified loci were verified by PCR and automated Sanger sequencing (Eurofins Genomics).

**Table 1.** Bacterial strains used in this study

Species	Description	Source/reference
***Escherichia coli***		
JM109	Cloning strain for generating plasmid constructs	Promega, U.K.
S17-1	Conjugative strain for transfer of plasmids to *Rba. sphaeroides*	Simon et al. [65]
***Rhodobacter sphaeroides***		
Wild type	Strain 2.4.1	S. Kaplan*
Δ*crtA*	Unmarked deletion of *crtA* (Rsp_0272); carotenoid pathway truncated at spheroidene	Chi et al. [50]
Δ*puyA*	Unmarked deletion of *puyA* (Rsp_7571); does not produce protein-Y	This study; Qian et al. [7]
Δ*puzA*	Unmarked deletion of *puzA* (orf located within Rsp_2385 gene); does not produce protein-Z	This study; Qian et al. [8]
Δ*olsBA*	Unmarked deletion of *olsBA* (Rsp_3826–3827); does not make ornithine lipids	This study; Aygun-Sunar et al. [49]
Δ*sqdB*	Unmarked deletion of *sqdB* (Rsp_2569); does not make SQDG	This study; Benning and Somerville [47]
Δ*crtA* Δ*puyA*	Unmarked deletion of *puyA* from Δ*crtA* background	This study
Δ*crtA* Δ*puzA*	Unmarked deletion of *puzA* from Δ*crtA* background	This study
Δ*crtA* Δ*olsBA*	Unmarked deletion of *olsBA* from Δ*crtA* background	This study
Δ*crtA* Δs*qdB*	Unmarked deletion of *sqdB* from Δ*crtA* background	This study
Δ*cycA* Δ*cycI*	Unmarked deletion of *cycA* (Rsp_0296) and *cycI* (Rsp_2577); does not produce cytochrome *c*_2_ (CycA) or isocytochrome *c*_2_ (CycI)	This study

* Department of Microbiology and Molecular Genetics, The University of Texas Medical School, Houston, TX 77030, U.S.A.

For complementation, plasmids were generated by amplifying fragments containing *sqdB* or *sqdBDC* with HindIII and BcuI restriction sites. The digested PCR products were ligated into a modified pBBRBB-P*puf*_843–1200_ plasmid [64] in which the *puf* promoter-*DsRED* fragment was replaced with the *pucBAC* genes and 364 bp upstream of *pucB*, corresponding to the *puc* promoter (P*puc*). A HindIII site was engineered immediately downstream of P*puc* such that *pucBAC* could be replaced with a gene of interest by HindIII-BcuI digestion. The *sqdB* and *sqdBDC* plasmids were conjugated into the Δ*sqdB* strain with selection on kanamycin-containing plates.

### Cell growth and preparation of intracytoplasmic membranes

*Rba. sphaeroides* cells were grown photoheterotrophically in 1 l Roux bottles containing M22 medium [66] under ∼50 µmol m^−2^ s^−1^ illumination from 70 W Phillips Halogen Classic bulbs until they reached stationary phase (an optical density at 680 nm [OD_680_] of ∼3). Strains containing pBBRBB plasmids were supplemented with 30 µg ml^−1^ kanamycin. Cells were harvested by centrifugation at 4000 RCF for 30 min at 4°C then resuspended in 5 ml 20 mM Tris–HCl pH 8. Following addition of a few crystals of DNaseI and lysozyme, cells were disrupted via two passes through a chilled French press at 18 000 psi followed by removal of unbroken cells and insoluble debris by centrifugation at 25 000 RCF for 30 min at 4°C. The supernatant was layered on top of 15/40% (w/v) discontinuous sucrose gradients and centrifuged at 85 000 RCF in a Beckman Type 45 Ti rotor at 4°C for 10 h. A pigmented band of ICM formed at the 15/40% interface, which was harvested using a serological pipette and stored at −20°C until required.

### TLC of lipids

TLC was performed based on a method by Swainsbury et al. [17] with some modifications. TLC plates were activated by soaking in 0.15 M ammonium sulfate for 15 min and placed in an oven at 160°C for 1 h. A 30 µl volume of each membrane fraction at an optical density at 875 nm (OD_875_) of 10 was dissolved in 100 µl of 50:50 methanol:chloroform, centrifuged in a benchtop microcentrifuge at 16 000 RCF for 5 min and the lower chloroform phase was removed. A volume of 5–10 µl of each lipid standard (∼5 mg ml^−1^ in chloroform) and membrane sample was loaded on to the TLC plate and run in 85:15:10:3.5 chloroform:methanol:acetic acid:water for 45 min. The plate was dried for 5 min before being submerged in 50% (v/v) H_2_SO_4_ for 10–20 s and dried, prior to heating at 160°C for 10 min to develop the lipid bands.

### Fractionation of photosynthetic complexes by rate-zonal centrifugation

Membranes harvested from discontinuous sucrose gradients were diluted at least 5-fold in 20 mM Tris-HCl pH 8 and pelleted at 185 000 RCF for 2 h using a Beckman Type 45 Ti rotor at 4°C. Pelleted membranes were resuspended in ∼100–200 μl of 20 mM Tris-HCl pH 8. Six OD_875_ units of resuspended membranes were solubilised in 3% (w/w) *n*-dodecyl-β-d-maltoside (β-DDM) in a total volume of 375 μl for 1 h at room temperature before centrifugation at 15 000 rpm at 4°C for 1 h in a microcentrifuge. The supernatant was collected and layered on top of discontinuous sucrose gradients containing steps of 20%, 21.25%, 22.5%, 23.75% and 25% (w/w) sucrose in 20 mM Tris–HCl pH 8 and 0.03% (w/v) β-DDM. Gradients were centrifuged in a Beckman SW41 Ti rotor at 125 000 RCF for 40 h at 4°C. Each gradient was performed in technical triplicate from two biological repeats. Pigmented bands were harvested with a peristaltic pump for downstream processing. If being used for turnover assays, RC-LH1 monomer and dimer bands were buffer exchanged into 50 mM Tris-HCl pH 7.5 with 100 mM NaCl and 0.03% (w/v) β-DDM by spin concentration with 50 000 MWCO centrifugal concentrators (Sartorius).

To attempt to reform RC-LH1 dimers from monomers of the Δ*sqdB* strain by provision of SQDG, the monomer band harvested from a discontinuous sucrose gradient of the Δ*sqdB* strain was concentrated to 500 µl in a 50 000 MWCO centrifugal concentrator (Sartorius). This sample was split and incubated with and without 0.05% (w/v) SQDG for 24 h before application to another discontinuous sucrose gradient, as described above.

### Purification of *Rba. sphaeroides* cytochrome *c*_2_

A cytochrome *c*_2_ overproduction strain was constructed by expressing *cycA* under the control of the constitutive P*puf_843–1200_* promoter on the pBBRBB plasmid in a strain lacking the genomic copies of *cycA* and *cycI*. Cell pellets from semi-aerobic cultures (1.6 l of medium in 2.5 l conical flasks shaken at 180 rpm at 30°C) were resuspended in periplasmic extraction buffer (100 mM Tris–HCl pH 8, 500 mM sucrose and 50 mM NaCl) supplemented with a tablet of EDTA-free protease inhibitor (Merck) up to a total volume of 40 ml. 0.8 g of solid sodium deoxycholate (Sigma) was added to the cell resuspension and, after an hour of incubation at 4°C in the dark, spheroplasts were pelleted at 30 000 RCF for 30 min at 4°C. The supernatant was transferred to a fresh centrifuge tube and 6.25 ml of deoxycholate precipitation solution (1 M ammonium acetate pH 5, 250 mM MgSO_4_) added, before the precipitate was removed by an identical centrifugation step. The supernatant from this second spin step was subsequently passed through two 0.22 µm filters (Sartorius) and made up to 500 ml with 50 mM ammonium acetate buffer at pH 5 before loading onto a 30 ml SP Sepharose column (Cytiva). Cation exchange was performed to purify the cytochrome using a gradient of 13–23% buffer B (50 mM ammonium acetate, 1 M NaCl).

### RC-LH1 turnover assays

Turnover assays were conducted under steady state conditions in a similar fashion to that described in Swainsbury et al. [33], using 300 µl solutions containing 5 µM reduced cytochrome *c*_2_, 50 µM ubiquinone-2 (Merck) and 0.01–0.05 µM RC-LH1 in a buffer mixture containing 50 mM Tris-HCl pH 8, 100 mM NaCl, 1 mM sodium D-ascorbate and 0.03% (w/v) β-DDM. Following overnight dark adaption, 300 µl of each reaction mixture were placed in a quartz cuvette and monitored at 550 nm using a Cary 60 spectrophotometer (Agilent Technologies). After 10 s, excitation energy was delivered via a fibre optic cable from an 810 nm M810F2 LED (light-emitting diode) (Thorlabs Ltd., U.K.) driven at 100% intensity using a DC2200 controller (Thorlabs Ltd., U.K.). The data were processed by fitting the linear initial rate over 0.025–0.1 s, starting from the first data point where the absorbance started dropping continuously. Rates were normalised to e^−^/RC/s by dividing the cytochrome *c*_2_ oxidation rate per second by the RC-LH1 concentration. The concentration of RC-LH1 complexes was determined using an extinction coefficient of 3000 mM^−1^ [67], except for the monomeric Δ*puyA* complex, which we predicted to have an extinction coefficient of 2835 mM^−1^ based upon spectra normalised to the RC bacteriopheophytin band in Figure 3E.

### Bioinformatics

Homologues of PufX, protein-Y, protein-Z and SqdB were identified by performing tblastn (protein to translated nucleotide) searches against the whole genome contigs (wgs) database on the NCBI BLAST webserver (https://blast.ncbi.nlm.nih.gov/Blast.cgi). Search parameters were calibrated using PufX as a benchmark, which is known to be highly divergent, and was complicated by its short sequence. The settings used to provide positive hits to all PufX proteins in the target species were: expect threshold = 20; word size = 2; max matches in a query range = 0 (default); matrix BLOSUM62 (default); gap costs = existence: 11, extension: 1 (default); compositional adjustments = no adjustment; filter low complexity regions = off. Searches were performed using the *Rba. sphaeroides* PufX sequence as a template (UniProt entry P13402) against each species individually. Top scoring hits for each species were verified by viewing relevant UniProt entries for each sequence, which were annotated as PufX in all cases. Next, searches for LH2 α (UniProt entry Q3J144), protein-Y (UniProt entry U5NME9), the resolved sequence of protein-Z extracted from PDB entry 7PQD, and SqdB (UniProt entry Q3J3A8) were performed using the same settings as for PufX. The top scoring hits from each species were verified by manually inspecting the genomes in the KEGG database (https://www.genome.jp/kegg/) or GENBANK, rejecting sequences embedded within larger genes or in unrelated genomic regions. LH2 hits were also verified via previous experimental determination of LH2 production in these species. A phylogenetic tree of 16S rRNA sequences was generated via a nucleotide BLAST of the *Rba. sphaeroides* 16S rRNA (Genbank entry KF791043.1) against the wgs database for all species analysed in this study, and the non-photosynthetic alphaproteobacterium *Caulobacter vibrioides* as an outgroup to root the tree. The tree was rendered using FigTree v1.44 (available at http://tree.bio.ed.ac.uk/software/figtree/) following flipping of some nodes to sort species by whether they form RC-LH1 dimers. We noted that *puyA* was located near to *otsB* (encoding trehalose-phosphatase) in the genome of the species that have this gene. Whole genome shotgun sequences of *Rba. changlensis* were searched manually for an ORF resembling *puyA* in the vicinity of *otsB.* The sequence in Supplementary Figure S7 was found in *Rba. changlensis* strain DSM 18774 NCBI Reference Sequence: NZ_QKZS01000001.1.

## Open Access

Open access for this article was enabled by the participation of University of East Anglia in an all-inclusive *Read & Publish* agreement with Portland Press and the Biochemical Society under a transformative agreement with JISC.

## Abbreviations

BChl: bacteriochlorophyll
cryo-EM: cryogenic electron microscopy
LED: light-emitting diode
LH2: light harvesting 2
RC-LH1: reaction centre-light harvesting 1
SQDG: sulfoquinovosyl diacylglycerol
TLC: Thin-layer chromatography
