# Supplementary material for: Sulfoquinovosyl diacylglycerol is required for dimerisation of the Rhodobacter sphaeroides reaction centre-light harvesting 1 core complex
Source: Biochem J. 2024 Jun 26;481(13):823–38. doi: 10.1042/BCJ20240125 (PMC11346425; doi:10.1042/BCJ20240125)
Supplement: Supplementary Material [file BCJ-481-823-s1.pdf]

Supporting information for Sulfoquinovosyl diacylglycerol is required for dimerization of the *Rhodobacter sphaeroides* RC-LH1 core complex

Elizabeth C. Martin[1], Adam G.M. Bowie[1], Taylor Wellfare Reid[1,3], C. Neil Hunter[1], Andrew Hitchcock[1,*] and David J.K. Swainsbury[2,*]

[1]Plants, Photosynthesis and Soil, School of Bioscience, University of Sheffield, Sheffield, UK.

[2]School of Biological Sciences, University of East Anglia, Norwich, UK.

[3]Present address: Centre for Bacterial Cell Biology, Newcastle University, Newcastle, UK.

*Corresponding authors: Andrew Hitchcock (a.hitchcock@sheffield.ac.uk) and David J.K. Swainsbury (d.swainsbury@uea.ac.uk)

Table of contents:

Table S1. Primers used in this study — Page 2

Table S2. Plasmids used in this study. — Page 3

Supplementary Figure 1. Growth curves — Page 3

Supplementary Figure 2. UV/Vis/NIR absorbance spectra of chromatophore membranes — Page 4

Supplementary Figure 3. Full TLC plate — Page 5

Supplementary Figure 4. Attempted reconstitution with SQDG — Page 6

Supplementary Figure 5. Confirmation of knockouts of *puyA*, *puzA, cycA* and *cycI* by PCR. — Page 7

Supplementary Figure 6. Additional sucrose gradients. — Page 7

Supplementary Figure 7. Sequence alignments for protein-Y and protein-Z — Page 8

Table S1. Primers used in this study. Restriction enzyme sites used for cloning are underlined in bold.

Name	Sequence (5′-3′)
olsBA KO Scr F	CTTTCCGAGATCAGCGCCATCTC
olsBA KO UF	GATC**GAATTC**CTGATAAGATCGTGACAGATGCGCG
olsBA KO UR	GACAGGCTCGTCGGCGATCATTCCCGGAC
olsBA KO DF	GATCGCCGACGAGCCTGTCGGCTGACCG
olsBA KO DR	GATC**AAGCTT**GATCGAGAACCATGTGCTGATGGTC
olsBA KO Scr R	GATCGATCTCGAGATCTTCCCCGAC
sqdB KO Scr F	CGGTGGGTGCCGACAAGAT
sqdB KO UF	GATC**GAATTC**GTGGCTGCCATCTGCCAT
sqdB KO UR	CCAATCAGGACACTGCGATGCGCATGAAGCC
sqdB KO DF	CGCATCGCAGTGTCCTGATTGGATCTGGCAG
sqdB KO DR	GATC**AAGCTT**AACAGCCGGTCCACGTTC
sqdB KO Scr R	TCTCGTAGACATTCGGCGCG
puzA KO Scr F	CATTTCTGCATCATCGCGCATGAC
puzA KO UF	CCG**GAATTC**GAAGCTGGACGAGATGTGGAATCC
puzA KO UR	CGTCAGACCTCTTTCATATATGCCATTTAAACCTCCCTCTTGC
puzA KO DF	GAGGTTTAAATGGCATATATGAAAGAGGTCTGACGGACCCGTG
puzA KO DR	CGGC**AAGCTT**CCATCGTTTTCGTCTTCCGTCAC
puzA KO Scr R	GTTCGACGATGGACAGGATCTCG
puyA KO Scr F	CAGCCGATGGTCCAGACCTC
puyA KO UF	CCG**GAATTC**GTCACGATAATGGGCCATGTCTCTC
puyA KO UR	CAGTTGCTGTTTTCGGGCATGGTGCCTCCTTC
puyA KO DF	CCATGCCCGAAAACAGCAACTGACGGCGC
puyA KO DR	CGGC**AAGCTT**GAGGGCTGGATCGACTACGATC
puyA KO Scr R	GGCCTATGTCTCGGGGTTTCTC
cycI KO Scr F	CATTTCGTGAATCCGTCCGAGATCG
cycI KO UF	CCG**GAATTCC**AACGTGAAGGTGATGCGTCAGG
cycI KO UR	CATTTCAGCCCTCCAATCTCATGGTCTTCTCCCTTTGCG
cycI KO DF	GACCATGAGATTGGAGGGCTGAAATGCCTGTCTGC
cycI KO DR	CTG**AAGCTT**GCCCACGTTCTCG
cycI KO Scr R	GCCACAGGATCTTGCCGTCATTG
cycA KO Scr F	CATGGTGGTGAACCTGCAGGAC
cycA KO UF	GCA**GAATTC**CCTCGCATCTGCCGGATACC
cycA KO UR	GGCGACCTGGGCCTTGACTTGGAACTTCATGG
cycA KO DF	GTCAAGGCCCAGGTCGCCGTCCGGC
cycA KO DR	CGC**AAGCTT**GGCGCCTGAATGTACTCACCG
cycA KO Scr R	CTGAAGCAGGCGGTGTCGG
sqdB HindIII F	GATC**AAGCTT**ATGCGCATCGCAGTTCTGG
sqdB BcuI R	GATC**ACTAGT**TCAGGACACCGAGCGCAG
sqdC BcuI R	GATC**ACTAGT**CTAAATCATGAGCGGCAGCGTTTG
sqdB seq F	GTTATCTCGACGTCTCGGTCGAGAC
sqdD seq F	GATTGGATCTGGCAGCCGAAGG
ECM 18	CCTACACGCAAACCGTCGATTTAC
PCR1	CGGGCCTCTTCGCTATT
PCR2	TTAGCTCACTCATTAGG

Table S2. Plasmids used in this study.

Name	Details	Source/reference
pk18mob*sacB*	Allelic exchange vector, Km^R	Professor J. Armitage*
pk18mob*sacB*::*ΔolsBA*	Construct for unmarked deletion of *olsBA*	This study
pk18mob*sacB*::*ΔsqdB*	Construct for unmarked deletion of *sqdB*	This study
pk18mob*sacB*::*ΔpuzA*	Construct for unmarked deletion of *puzA*	This study
pk18mob*sacB*::*ΔpuyA*	Construct for unmarked deletion of *puyA*	This study
pk18mob*sacB*::*ΔcycA*	Construct for unmarked deletion of *cycA*	This study
pk18mob*sacB*::*ΔcycI*	Construct for unmarked deletion of *cycI*	This study
pBBRBB-P*puf*$_{843\text{-}1200}$-DsRed	Replicative expression plasmid, Km^R	Addgene.org; Tikh *et al.* 2014 [48]
pBBRBB-P*puf*$_{843\text{-}1200}$-*cycA*	Plasmid for expression of *cycA*	This study
pBBRBB-P*puc-pucBAC*	pBBRBB-P*puf*$_{843\text{-}1200}$-DsRed with P*puf-D*sRED replaced with the P*puc-pucBAC*	This study
pBBRBB-P*puc-sqdB*	Plasmid for expression of *sqdB*	This study
pBBRBB-P*puc-sqdBDC*	Plasmid for expression of *sqdBDC*	This study

*Department of Biochemistry, University of Oxford, South Parks Road, Oxford OX1 3QU, U.K.

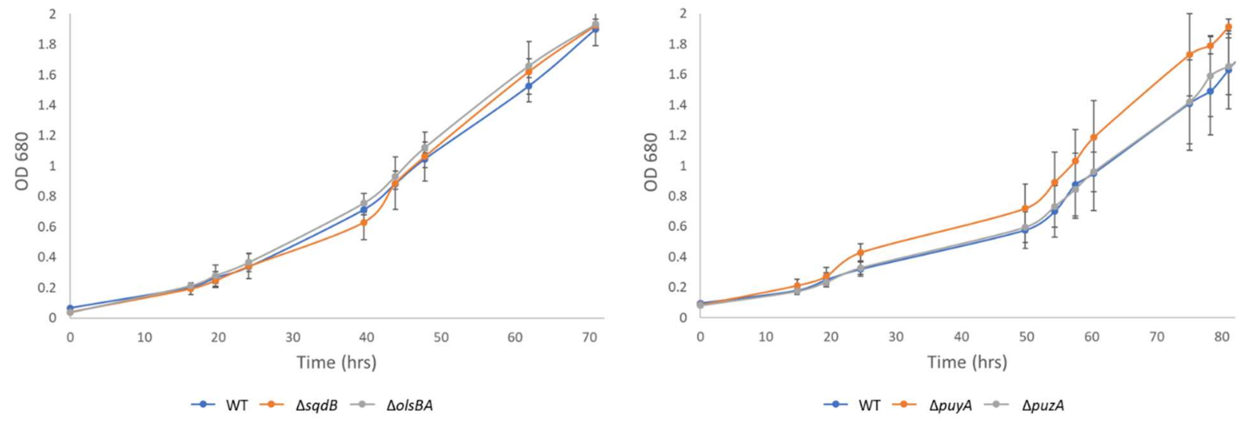

Supplementary Figure 1. Growth curves of all four knockouts in a WT background used in this study at low light (10 µmol). No significant phenotype was observed in any strain and all knockouts were confirmed by PCR afterwards. Δ*puyA* showed some variation, but further repeats (data not shown) showed the same growth as WT. A phenotype may yet be apparent at different light intensities.

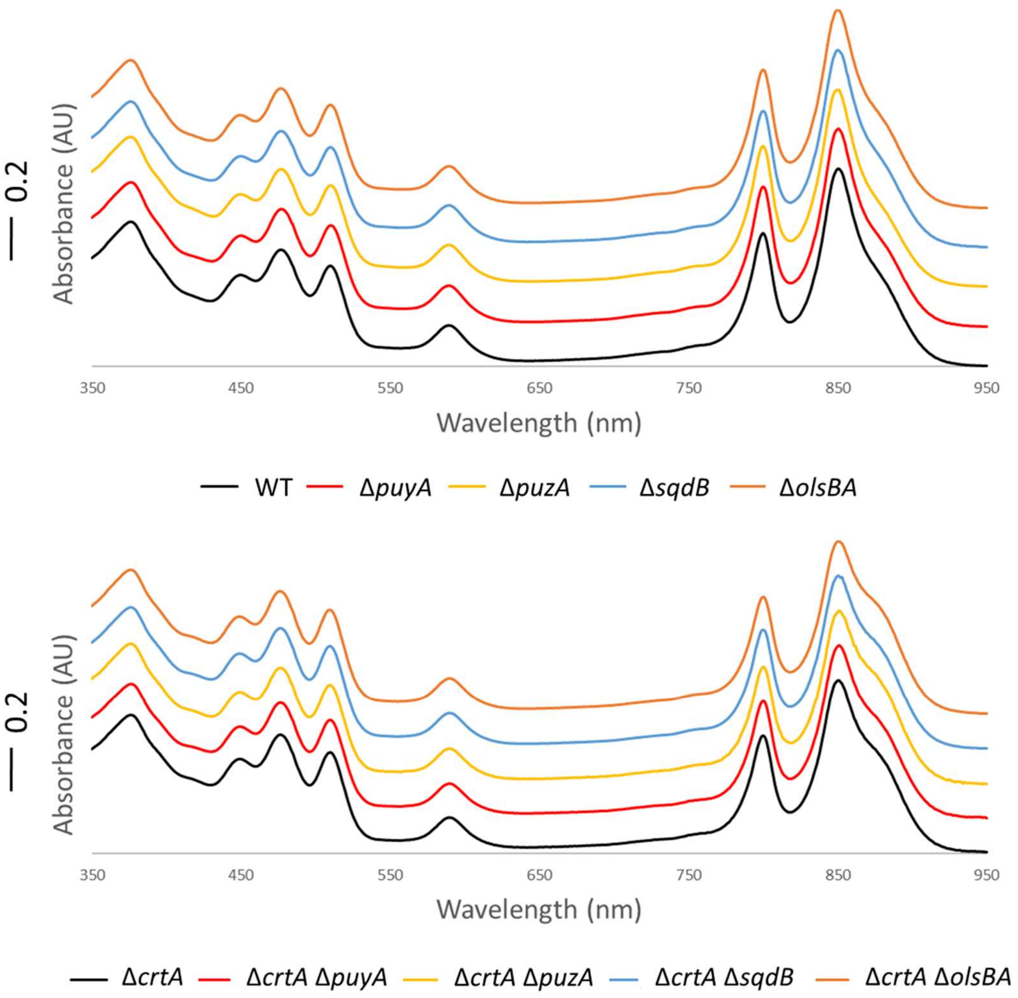

Supplementary Figure 2. UV/Vis/NIR absorbance spectra of chromatophore membranes from all strains in the WT and Δ*CrtA* backgrounds. Spectra collected of chromatophore membranes isolated from other cellular components by differential centrifugation (see methods). Spectra are offset for clarity.

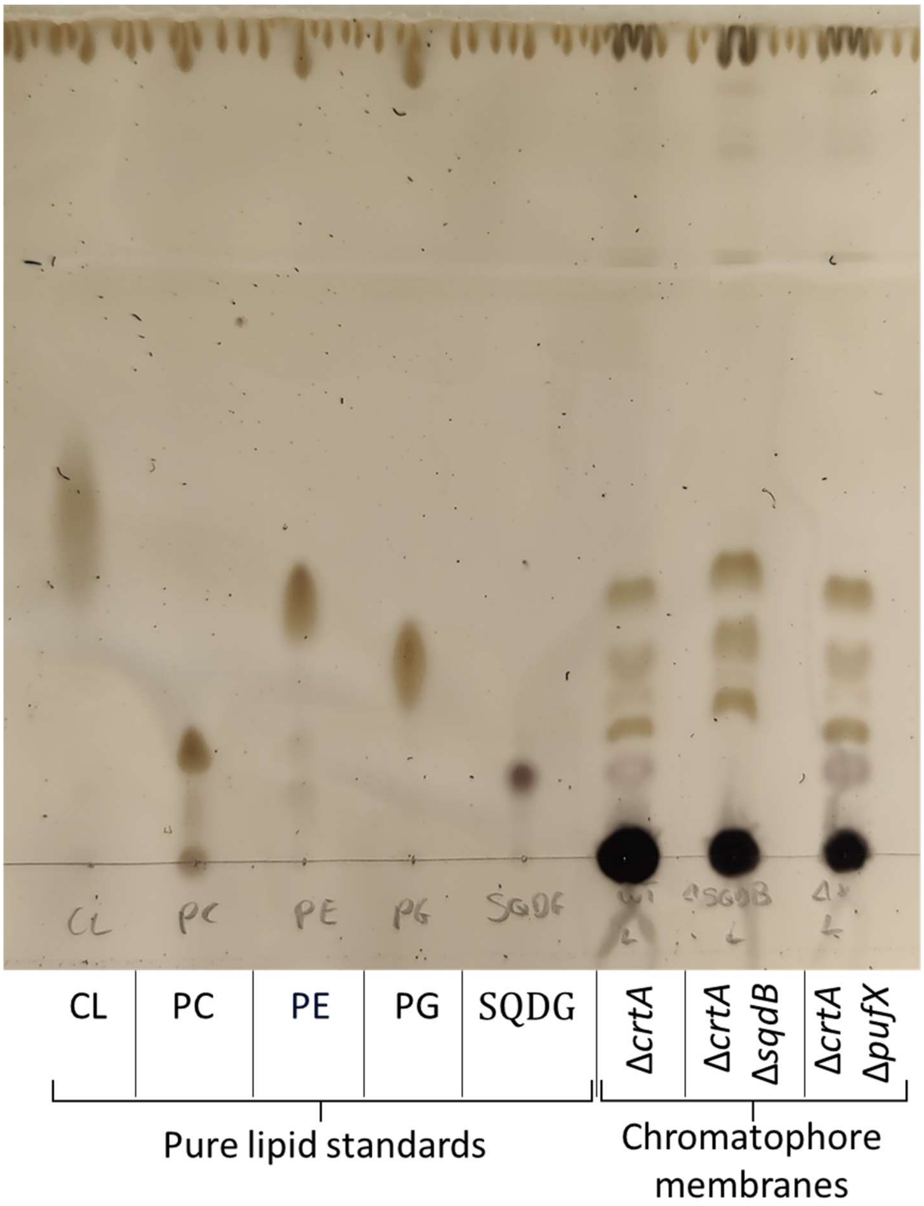

Supplementary Figure 3. Full TLC plate showing pure lipid standards and lipids extracted from chromatophore membranes. The lipid standards were Cardiolipin (CL), Phosphatidylcholine (PC), Phosphatidylethanolamine (PE), Phosphatidylglycerol (PG), Sulfoquinovosyl diacylglycerol (SQDG). Chromatophore membranes were extracted from *Rba. sphaeroides* cells and isolated by separation on 40/15 % w/v sucrose gradients from the Δ*crtA*, Δ*crtA* Δ*sqdB*, and Δ*crtA* Δ*pufX* strains.

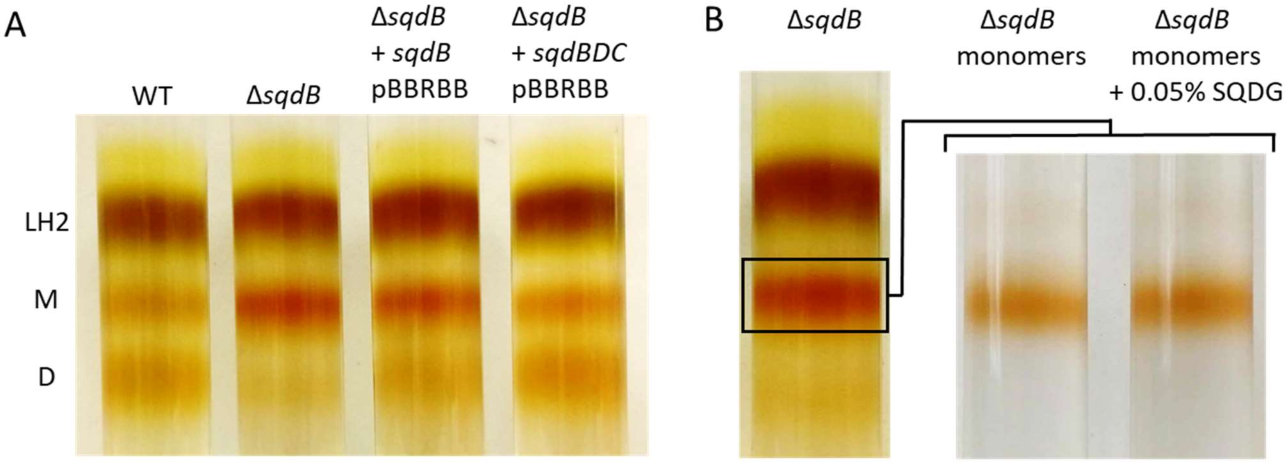

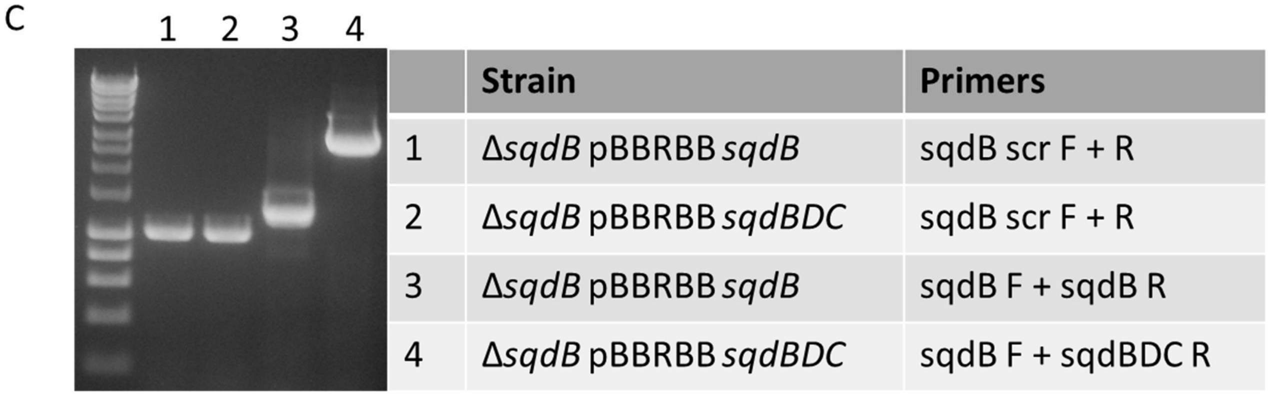

	Strain	Primers
1	Δ*sqdB* pBBRBB *sqdB*	sqdB scr F + R
2	Δ*sqdB* pBBRBB *sqdBDC*	sqdB scr F + R
3	Δ*sqdB* pBBRBB *sqdB*	sqdB F + sqdB R
4	Δ*sqdB* pBBRBB *sqdBDC*	sqdB F + sqdBDC R

Supplementary Figure 4. Attempted reconstitution of dimers in the Δ*sqdB* strain by *in trans* complementation or incubation with SQDG. (A) Monomer-dimer gradients of Δ*sqdB* cells expressing *sqdB* from a plasmid show a slight increase in dimer formation. Expression of the *sqdBDC* operon increases dimer expression to WT levels. (B) Purified monomers from Δ*sqdB* incubated with purified SQDG do not spontaneously form dimers. (C) Ethidium bromide-stained PCR products to verify the presence of *sqdB* or *sqdBDC* in pBBRBB-Ppuf$_{843\text{-}1200}$ in the Δ*sqdB* background. Lanes 1 and 2 confirm the absence of *sqdB* in the genome of both strains and lanes 3 and 4 confirm the presence of either *sqdB* or *sqdBDC* on pBBRBB.

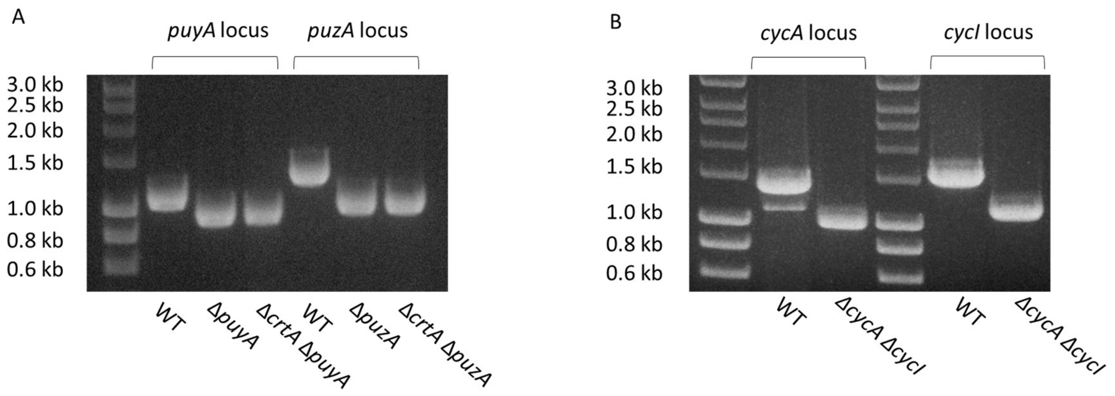

Supplementary Figure 5. Confirmation of knockouts of *puyA*, *puzA, cycA* and *cycI* by PCR. Agarose gel of ethidium bromide-stained PCR products showing size differences for the amplified regions. A. PCR products spanning the *puyA* and *puzA* genes showing a clear reduction in size in the knockout strains relative to the wild type. B. PCR products spanning the *cycA* and *cycI* genes showing a clear reduction in size in the knockout strains relative to the wild type.

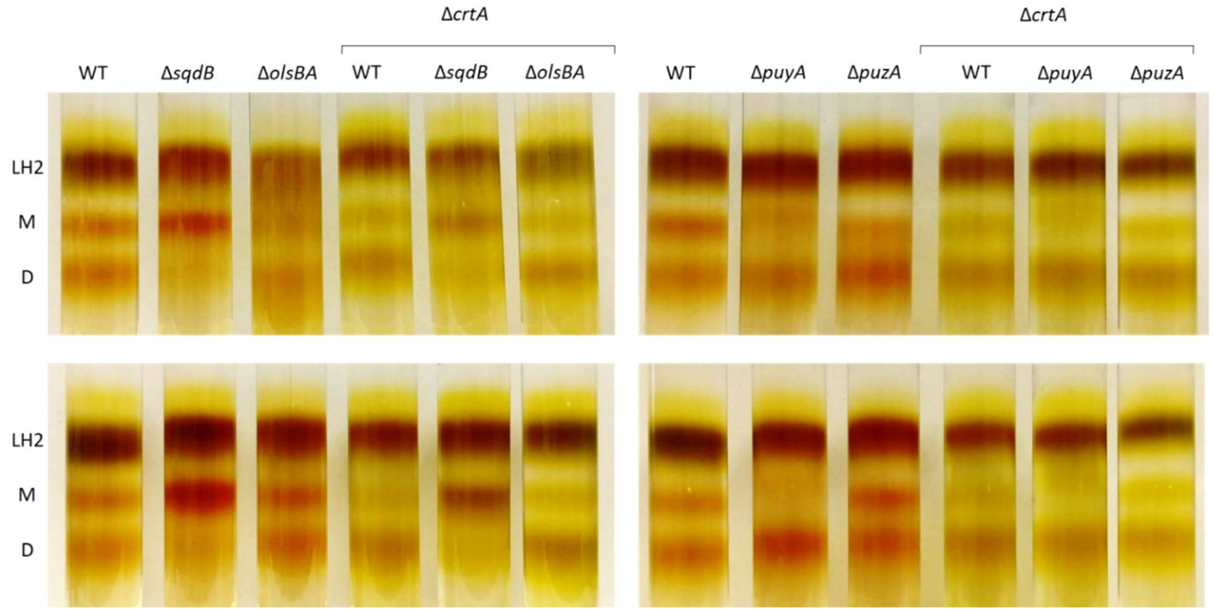

Supplementary Figure 6. Additional sucrose gradients. Two further repeats were performed showing the same monomer dimer distribution as presented in Figure 3 of the main paper.

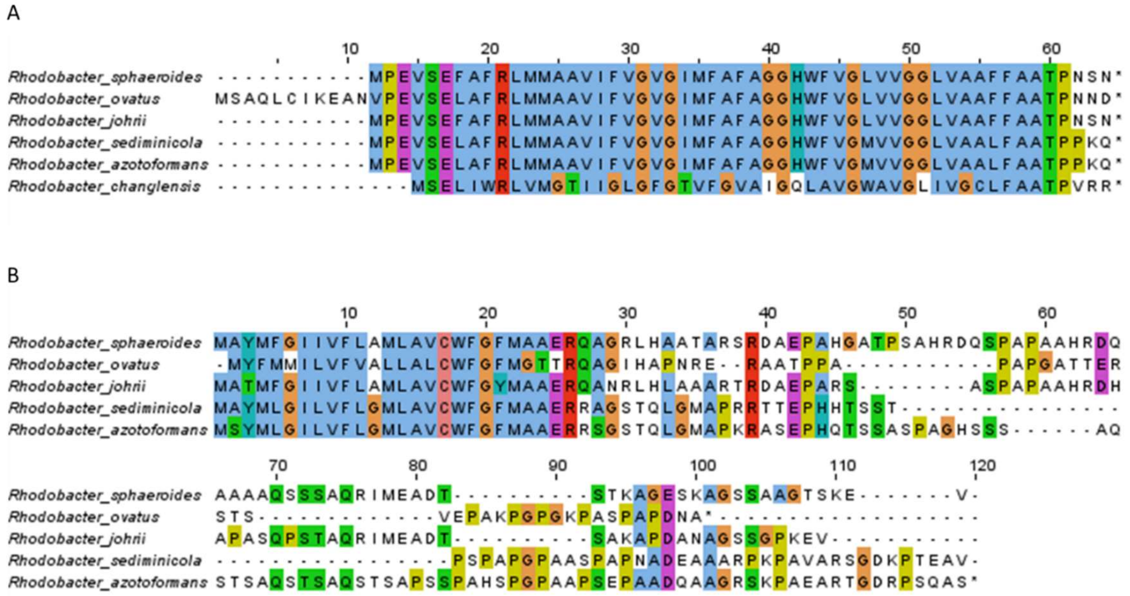

Supplementary Figure 7. Sequence alignments for protein-Y and protein-Z (A) Alignment of sequences for protein-Y from species within the *Cereibacter* subgroup. (B) Alignments for protein-Z. A sequence for *Rba. changlensis* could not be found, potentially due to a lack of homology. With the exception of *Rba. changlensis*, protein-Y shows a very high degree of sequence homology between species. Protein-Z has a disordered tail on the C-terminus that is missing in the structure and shows a very high degree of variation between species. Truncations would have to be performed to establish if this region is unnecessary.
